# Fermentation Process and Metabolic Flux of Ethanol Production from the Detoxified Hydrolyzate of Cassava Residue

**DOI:** 10.3389/fmicb.2017.01603

**Published:** 2017-08-22

**Authors:** Xingjiang Li, Yongdong Deng, Ying Yang, Zhaojun Wei, Jieshun Cheng, Lili Cao, Dongdong Mu, Shuizhong Luo, Zhi Zheng, Shaotong Jiang, Xuefeng Wu

**Affiliations:** ^1^Department of Biological Engineering, School of Food Science and Engineering, Hefei University of Technology Hefei, China; ^2^Key Laboratory for Agricultural Products Processing of Anhui Province, Hefei University of Technology Hefei, China; ^3^Department of Environment Engineering, School of Environment and Energy Engineering, Anhui Jianzhu University Hefei, China

**Keywords:** *Candida tropicalis*, fermentation, xylose, ethanol, oxygen

## Abstract

With the growth of the world population, energy problems are becoming increasingly severe; therefore, sustainable energy sources have gained enormous importance. With respect to ethanol fuel production, biomass is gradually replacing grain as the main raw material. In this study, we explored the fermentation of five- and six-carbon sugars, the main biomass degradation products, into alcohol. We conducted mutagenic screening specifically for *Candida tropicalis* CICC1779 to obtain a strain that effectively used xylose (*Candida tropicalis* CICC1779-Dyd). By subsequently studying fermentation conditions under different initial liquid volume oxygen transfer coefficients (*k*_L_α), and coupling control of the aeration rate and agitation speed under optimal conditions, the optimal dissolved oxygen change curve was obtained. In addition, we constructed metabolic flow charts and equations to obtain a better understanding of the fermentation mechanism and to improve the ethanol yield. In our experiment, the ethanol production of the wild type stain was 17.58 g·L^−1^ at a *k*_L_α of 120. The highest ethanol yield of the mutagenic strains was 24.85 g·L^−1^. The ethanol yield increased to 26.56 g·L^−1^ when the dissolved oxygen content was optimized, and the conversion of sugar into alcohol reached 0.447 g·g^−1^ glucose (the theoretical titer of yeast-metabolized xylose was 0.46 g ethanol/g xylose and the glucose ethanol fermentation titer was 0.51 g ethanol/g glucose). Finally, the detected activity of xylose reductase and xylose dehydrogenase was higher in the mutant strain than in the original, which indicated that the mutant strain (CICC1779-Dyd) could effectively utilize xylose for metabolism.

## Introduction

With the world population growing and available land becoming scarce, energy problems are an increasing concern. Consequently, the search for alternatives to oil is a major direction of development, with ethanol derived from biomass gradually being explored by different countries (Quintero et al., 2013). For many years, grain has been the main raw material for fermented ethanol; however, reliance on grain has become a serious issue (Duque et al., 2015). In rural areas, with the costs of grain soaring, the production of ethanol from the fermentation of grain is becoming unsustainable leading to an urgent need for a cheap raw material replacement. In this respect, the biotransformation of cellulose is of great significance and could help to address the current worldwide energy crisis (Ebrahimi et al., 2017), grain shortages, and environmental pollution. Cellulosic and hemicellulose-rich biomass is the most promising ethanol feedstock, especially as fibrous raw materials are the richest renewable resources on Earth. The composition of cellulosic and hemicellulose-rich biomass is generally 31–40% cellulose, 35–48% hemicellulose, and 15–25% lignin. The use of lignocellulosic biotransformation to produce ethanol is currently a rapidly expanding area of research, and the effective use of xylose and glucose is one of the key aspects to making this biotransformation industrially viable. In other studies, sugar cane and wheat straw were both used for the production of alcohol (Dias et al., 2013; Tomas-Pejo et al., 2017). China's annual use of raw fibrous materials is about 7 × 10^8^ t, primarily from agriculture, forestry, industry, and urban waste (Alves et al., 2015; Kang et al., 2015; Khare et al., 2015). These fibrous waste products may be used as raw materials in the production of alcohol. The main product of cellulose hydrolysis is glucose and other six-carbon sugars, while hemicellulose hydrolyzatesare mainly xylose and other five-carbon sugars (Brienzo et al., 2009; Kamoldeen et al., 2017).

Cassava residue, a waste of lignocellulose, is the remaining solid waste after the production of tapioca starch. Its main components include starch, cellulose, hemicellulose, lignin, and a small amount of protein. It has a great recycle value and is consistent with sustainable development strategies; in particular, and it would reduce the waste of resources (Cheng et al., 2008, 2015).

Studies revealed that bacteria, filamentous fungi, and yeasts could produce alcohol by xylose fermentation. Bacteria can use several types of sugar for ethanol fermentation, but the rate is very low with the generation of many by-products. Fungi are mainly suitable for the simultaneous saccharification and fermentation of plant fiber raw materials, but the fermentation period is too long. Among those microorganisms for usage with xylose, yeast has the strongest fermentation capacity. In terms of the oxygen demand, this process can be explained by one of the two models: (1) anaerobic fermentation or (2) aerobic fermentation. Candida fits into the latter category (Mattam et al., 2016). Most studies have considered three commonly used yeast strains with pentose as a fermentation material for industrial application: *Pachysolen tannophilus, Pichia stipites*, and *Candida tropicalis*. Harinder used *Candida tropicalis* for ethanol production from rice straw, and the result showed that this strain could adapt to wood hydrolysate, with the yield reaching 77% of its theoretical value (Harinder et al., 2010).

Furthermore, the effects of dissolved oxygen on the fermentation of yeast have been studied. Appropriate amount of dissolved oxygen is found to be an important control parameter for ethanol fermentation; in particular, it strongly influences redox balance, cell growth, and energy generation for xylose transport (Liang et al., 2014).

If complete use of xylose from a raw fibrous material could be achieved, alcohol production could be increased by 25%. To realize the use of raw fibrous materials for the industrial production of alcohol, the effective use of both five- and six-carbon sugars is of great importance (Galbe and Zacchi, 2012).

## Materials and methods

### Screening for mutants

Slant medium (1 L) was prepared by dissolving glucose (20 g), yeast extract (10 g), peptone (20 g), and agar (20 g) in water. Liquid medium (1 L) was prepared by dissolving glucose (20 g), yeast extract (10 g), and peptone (20 g) in water. The screening medium (1 L) was prepared by dissolving xylose (70 g), yeast extract (10 g), peptone (20 g), and agar (20 g) in water. The fermentation medium (1 L) was prepared by dissolving including glucose (30 g), xylose (30 g), ammonium sulfate (5 g), potassium dihydrogen phosphate (1 g), yeast extract (10 g), magnesium sulfate heptahydrate (1 g), and peptone (20 g) in water.

*Candida tropicalis CICC1779* was cultured to the logarithmic growth phase, and then the yeast concentration was adjusted with physiological saline to 10^9^ cfu·mL^−1^. Under the action of a magnetic stirrer, the yeast was irradiated with UV light for 20–80 s (Watanabe et al., 2011). Dilute mutated yeast solutions were coated onto a solid plate, inoculated in the xylose screening medium, and incubated at 30°C for 2 days. Then, selected well-grown strains were inoculated into high concentration xylose screening medium for 1–2 days. At last, using the Dushi tube screening method, tetrazolium chloride (TTC) was used as a colorimetric method for screening yeast mutants. TTC is a colorimetric indicator, which is originally colorless but turns red in the presence of live yeast with strong dehydrogenase reduction activity. Thus, in the presence of TTC, the tiny colonies on the plate become brightly dyed with a visible red color. The color intensity is an indicator of the yeast's capacity to produce alcohol, with deep red indicating high alcohol production, followed by pink for moderate production, and colorless for little or no production (Guo et al., 2016). After screening, the strain that yielded the best alcohol production and was the most robust was chosen and named *Candida tropicalis CICC1779- Dyd* (deposited at Hefei University of Technology).

### Preparation of seed liquid

Liquid medium was placed in an autoclave at 121°C for 20 min and then cooled to room temperature. The yeast was stored in a liquid medium in a test tube at 4°C. To prepare the seed liquid, the yeast was placed on a shaker (120 r·min^−1^, 34°C) for 13 h, and inoculated into fresh liquid medium at 10% inoculums for 13 h. Finally, samples were centrifuged at 7,000 g for 8 min. The supernatant was discarded, and the suspension was supplemented with physiological saline.

### Fermentation process

The effects of different liquid oxygen transfer coefficients (*k*_L_α) on fermentation were examined. The main factors that determine the *k*_L_α value are speed and ventilation (Garcia-Ochoa et al., 2009). The fermentation medium (7 L) was added to a 15 L boiling tank (Sartorius, Gottingen, Germany). The sterilization conditions were 121°C for 15 min. After sterilization, samples were cooled to 34°C, followed by inoculation of the yeast suspension. The fermentation temperature was set to 34°C and the initial pH was adjusted to 4.0. This experiment used a two-factor three-level central composite design (CCD; Design Expert 8.0, USA) in Table 1.

**Table 1:** Central composite experimental design.

**Experiments**	**Coded levels**		**Real levels**	
	**X_1_**	**X_2_**	**X_1_ (rpm)**	**X_2_ (L·min^−1^)**
1	1	1	450	3
2	−1	1	150	3
3	1	−1	450	1
4	−1	−1	150	1
5	0	0	300	2
6	0	0	300	2
7	0	0	300	2

*A, Agitation speed; B, Aeration rate*.

### Analytical methods

The sugar content was determined by high-performance liquid chromatography (Agilent 1260; Santa Clara, CA, USA) using an HPX-87 sugar analysis column. The mobile phase was 5 mmol·L^−1^ H_2_SO_4_ with a solution flow rate of 0.6 mL·min^−1^. The column temperature was 65°C (Morales et al., 2015).

The ethanol content was determined by gas chromatography (Agilent 6890N) using the DB-624 column. The column temperature was 100°C for 1 min then increased at a rate of 15°C·min^−1^ to reach 190°C, which was maintained for 3 min. The column flow rate for high purity nitrogen was 30 mL·min^−1^ (constant current). The gas flow was comprised of hydrogen at 300 mL·min^−1^ and air at 400 mL·min^−1^. The inlet temperature was 200°C and the detector temperature was 250°C (Dong et al., 2013). The cell weight was measured by drying at a constant temperature.

### Determination of the oxygen transfer coefficient (*k*_L_α)

The initial volumetric oxygen transfer coefficient (*k*_L_α) was determined using the dynamic gassing-out methodology (physical method). Nitrogen was used to reduce the dissolved oxygen content to zero before fermentation, and the agitation speed and aeration rate were immediately adjusted to the same conditions used in the fermentation experiments. The dissolved oxygen concentration was measured at 5 s intervals throughout the aeration process using a serializable galvanic electrode (INPRO 6800 series; Mettler-Toledo, Columbus, OH, USA) connected to a Teflon-silicone-Teflon membrane. The equipment was previously calibrated at atmospheric pressure. The dissolved oxygen mass balance in the liquid phase can be expressed as follows (Fernández-Sandoval et al., 2017):

(1) $$\frac{{\text{dC}}_{\text{L}}}{d}=k_{\text{L}}\alpha \left({\text{C}}^{*}-{\text{C}}_{\text{L}}\right)-{\text{r}}_{\text{o2}}=\text{OTR}-\text{OUR}$$

Where C_L_ represents the dissolved oxygen concentration, C^*^ is the saturated oxygen concentration in the liquid, and OTR is the rate of oxygen transfer. When the oxygen uptake rate (OUR) is zero, the mass balance in the oxygen liquid phase can be simplified as follows:

(2) $$\frac{{\text{dC}}_{\text{L}}}{d}=k_{\text{L}}\alpha \left({\text{C}}^{*}-{\text{C}}_{\text{L}}\right)-{\text{r}}_{\text{o2}}=\text{OTR}$$

The initial *k*_L_α value was obtained based on the straight-line slope representing the oxygen mass balance integration in the absence of microorganisms:

(3) $$\text{ln}(1-\frac{{\text{C}}_{\text{L}}}{{\text{C}}^{*}})=-k_{L}\alpha \times \text{t}$$

### Metabolic flow analysis method

The metabolic flux analysis was carried out with the intention of calculating volumetric rates of formation of intracellular metabolites (Nielsen, 2001). On the basis of a pseudo-steady-state assumption for intracellular metabolites (Riascosa et al., 2005), there was no accumulation of any intermediates:

(4) $$r={\text{G}}^{\text{T}}\text{V}$$

Where r represents the net formation rates of metabolites (mmol/g DW h), V is the internal reaction rates (mmol/g DW h), and G is the total stoichiometric matrix for all reactants and products of reactions. Finally, the metabolic flux calculation was carried out using the mathematical functions of MMULT and MINVERSE in EXCEL 2007.

### Data analysis

All values were measured in triplicate with their uncertainty within 5%. One-way analysis of variance (ANOVA) with Duncan's new multiple range test was used (*p* < 0.05). All analyses were performed using Microsoft Office Excel 2007 and Origin 8.0 (OriginLab USA).

### Preparation of hydrolyzed sugar from cassava residue

First, the appropriate amount of cassava residue was weighed and placed in a cooking pot (Dussán et al., 2014). Then, the appropriate amount of 0.8% H_2_SO_4_ was added and the mixture was heated to 190°C in an oil bath, undergoing hydrolysis conditions for 4 min before removal and rapid cooling. The sample was then centrifuged to obtain a primary hydrolyzate (Souto et al., 2017). The remaining residue was placed into the cooking pot, and the appropriate amount of 1.8% H_2_SO_4_ was added. The mixture was heated at 220°C in an oil bath for 4 min, after which it was removed and rapidly cooled. After centrifugation, the secondary hydrolyzate was collected (Nanssou et al., 2016). The two hydrolyzate fractions were mixed. Excess calcium hydroxide was added to the hydrolyzate, mixed well, and the mixture was then centrifuged at 8,000 r·min^−1^ for 10 min. The supernatant was obtained, and its pH was adjusted to 5.0 with dilute sulfuric acid. After this, the appropriate amount of activated carbon was added for detoxification and certain concentration of fermented sugar was obtained accordingly.

## Results and discussion

### Preliminary analysis of mutant strains

**Figure 1:**
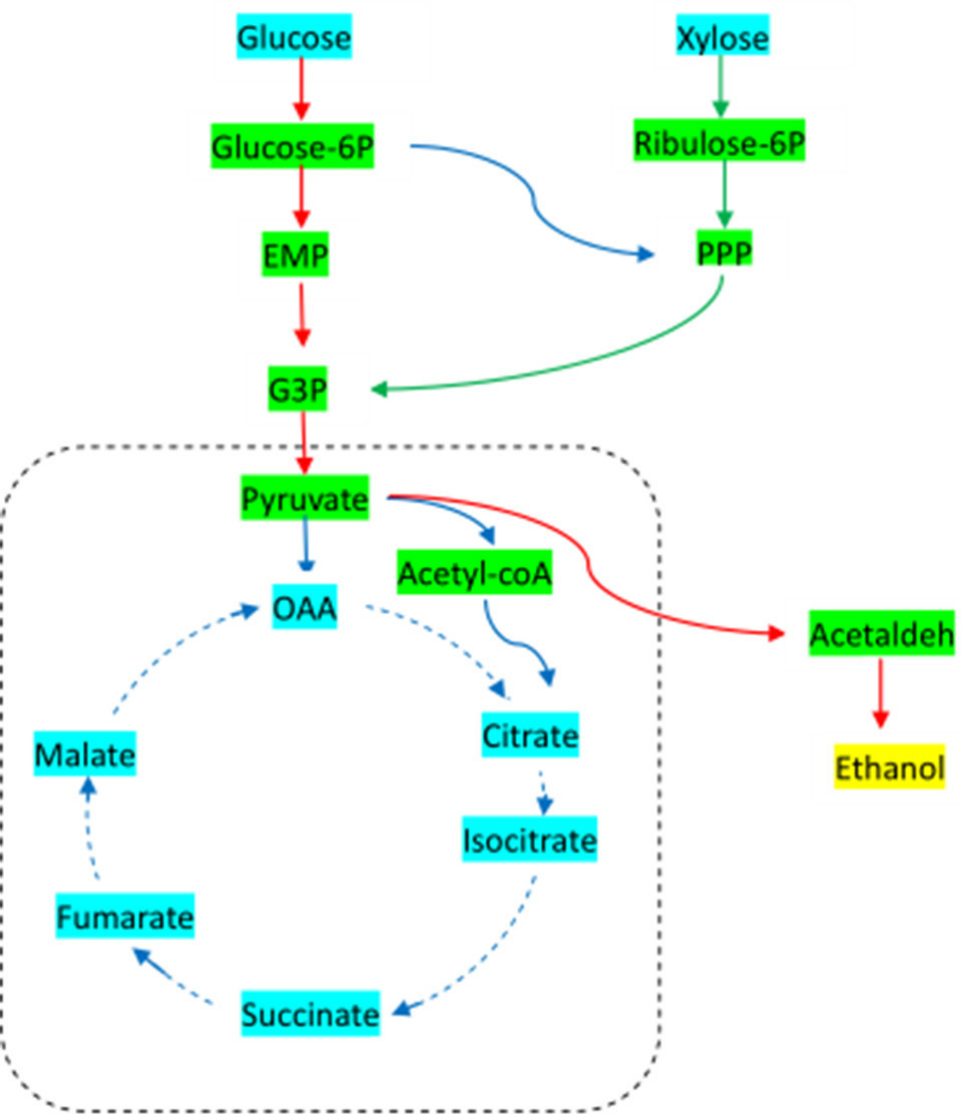
Basic metabolic pathway for *Candida tropicalis CICC1779*-*Dyd*. EMP, embden-meyerhof-parnas pathway; PPP, pentose phosphate pathway; G3P, glyceraldehyde 3-phosphate; OAA, oxaloacetic acid.

The basic metabolic pathway analysis of yeast was showed in Figure 1. Under aerobic conditions, yeast goes through the EMP pathway to obtain pyruvate, and from the pyruvate enters into the TCA cycle, resulting in the energy used to maintain the growth and metabolism of cells. In the absence of oxygen, pyruvic acid is converted directly to acetaldehyde, and then converted to ethanol, which is essentially the anaerobic respiration pathway of yeast. Xylose, on the other hand, is first transformed by xylose reductase to get xylitol, and then is converted by xylose dehydrogenase to xylulose. Finally, the products go through the phosphate pentose pathway (PPP), which is involved in the EMP pathway, in order to complete the use of five- and six-carbon sugars. The key enzymes of these processes include xylose reductase, xylose dehydrogenase, pyruvate decarboxylase, and ethanol dehydrogenase.

**Figure 2:**
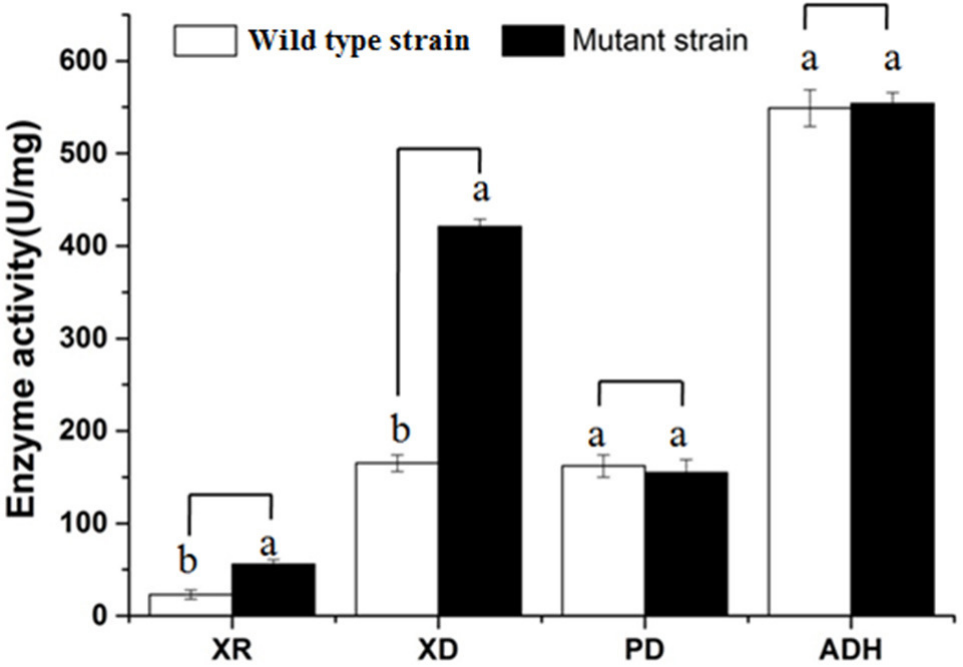
Comparison of key enzyme activities. XR, Xylose dehydrogenase; XD, Xylose dehydrogenase; PD, Pyruvate decarboxylase; ADH, Alcohol dehydrogenase; a, b the bars show significant differences (*P* < 0.05).

As can be seen from Figure 2, the activities of several key enzymes differ between the original yeast strain and the mutant strain. We can see significant improvement in the activity of the mutant strains of xylose reductase and xylose dehydrogenase relative to the wild type stain, indicating that the existence of metabolic xylose-related enzymes leads to the effective use of xylose for metabolism, in preparation for the coupling of the PPP path with the EMP path. From the high activity of pyruvate decarboxylase and ethanol dehydrogenase, it is evident that the yeast has a strong ability to produce alcohol.

### Fermentation results for various initial *k*_L_α values (72 h)

In order to investigate the fermentation potential of the mutant strain, we studied on its fermentation parameters and the results were showed in Tables 2, 3.

**Table 2:** Test coding and fermentation results.

**No**.	**Variables**		**K_L_α initial (h^−1^)**	**Results**	
	**X_1_ (rpm)**	**X_2_ (L·min^−1^)**		**Ethanol (g/L)**	**Cell concentration (g/L)**
1	450	3	145	21.23	6.12
2	150	3	100	23.01	5.79
3	450	1	120	24.85	6.49
4	150	1	55	18.45	5.45
5	300	2	85	22.58	5.87
6	300	2	86	22.57	5.86
7	300	2	84	22.56	5.84

**Table 3:** Parameter estimates for fermentation results for various initial *k*_L_α values (72 h).

**Parameters**	**K_L_α initial (h^−1^)**				
	**55**	**85**	**100**	**120**	**145**
Residual concentration of glucose (g/L)	1.35	1.22	3.56	2.35	2.98
Residual concentration of xylose (g/L)	1.32	1.21	3.21	2.05	2.58
Cell concentration (g/L) (g/L)	5.45	5.86	5.79	6.49	6.12
Ethanol titer (g/L)	18.45	22.58	23.01	24.85	21.23
Ethanol productivity Q_P_ (g/L·h^−1^)	0.256	0.314	0.320	0.345	0.296
Biomass yield Y_x/s_ (g/g)	0.095	0.102	0.109	0.117	0.112
Ethanol yield Y_p/s_ (g/g)	0.322	0.392	0.432	0.447	0.390

Extensive research and production practice indicates that the factors that affect the *k*_L_α value are agitation speed, aeration rate, physicochemical properties of the fermentation broth, foam state, shape of the air distributor, and the structure of the fermentor. However, we found the main factors were agitation speed and aeration rate (Table 2). The higher the agitation speed and aeration rate, the higher the *k*_L_α value. The stirrer in the fermentor maintains a uniform temperature and nutrient concentration in the fermentor, dispersing the air introduced into the fermentation broth in small bubbles to increase the gas–liquid contact area and strengthen the turbulence of the fermentation broth, thereby increasing the *k*_L_α value. An increased aeration rate will increase *k*_L_α, but when it is too large, the agitator exhibits the “air pan” phenomenon, and *k*_L_α no longer increases (Kirk and Szita, 2013). An aeration rate that is too large is not conducive to the dispersion and maintenance of air in the tank, leading to a concentration of the fermentation solution, which affects the transmission of oxygen. However, when the aeration rate is too low, metabolic emissions cannot be discharged quickly, and oxygen transfer is affected.

After 72 h of fermentation under each condition, the parameter values for broth were summarized in Table 3. When *k*_L_α was 120, the ethanol titer reached 24.58 g·L^−1^, the conversion of sugar into alcohol was 0.447 g·g^−1^, and the dry weight of the cells was 6.49 g·L^−1^. The lowest *k*_L_α was 55, which gave an ethanol titer of 18.45 g·L^−1^, a sugar conversion rate of only 0.322 g·g^−1^, and a cell dry weight of 5.45 g·L^−1^. This indicates that *k*_L_α has a significant impact on ethanol production, and if it is too high or too low, ethanol production will be inhibited_._

**Figure 3:**
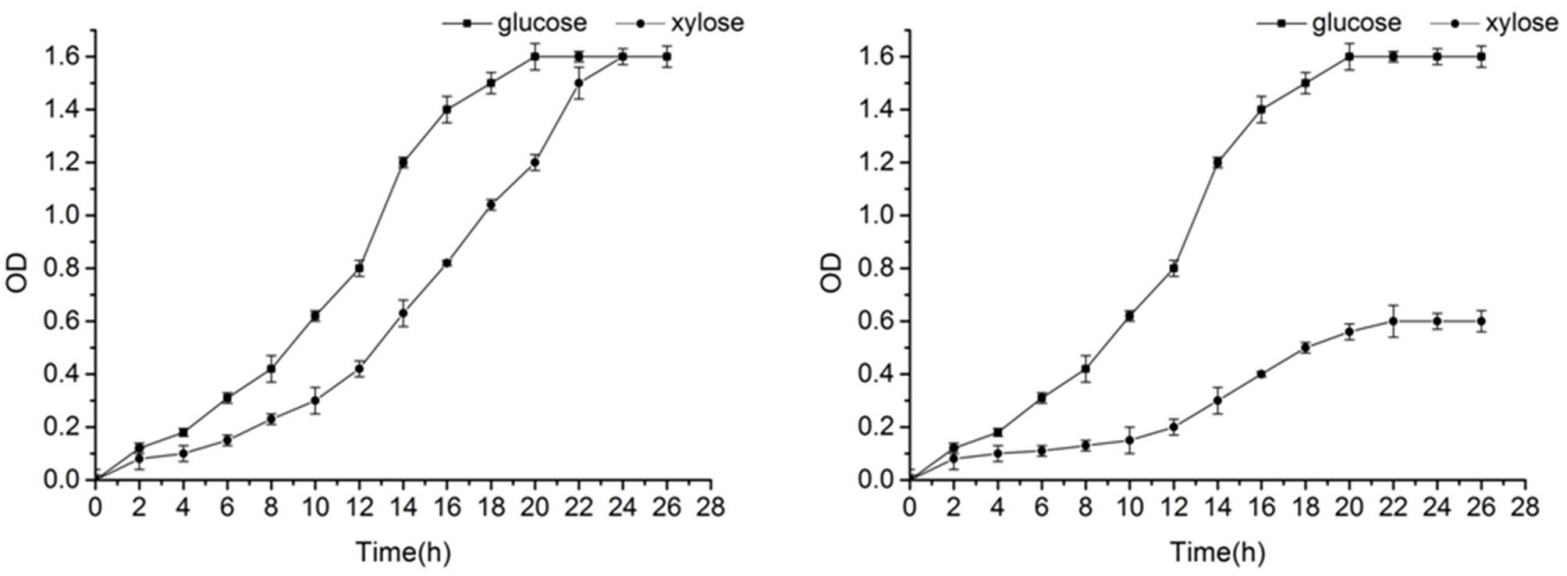
Growth curve of *Candida tropicalis CICC1779–Dyd* **(left)** and wild type strain **(right)**.

The yeast growth curve was showed in Figure 3, which demonstrates that the yeast can grow well in glucose and xylose. The growth curve showed a good S-shape, reaching its logarithmic growth phase, the point when cell growth is highest, at 13 h.

**Figure 4:**
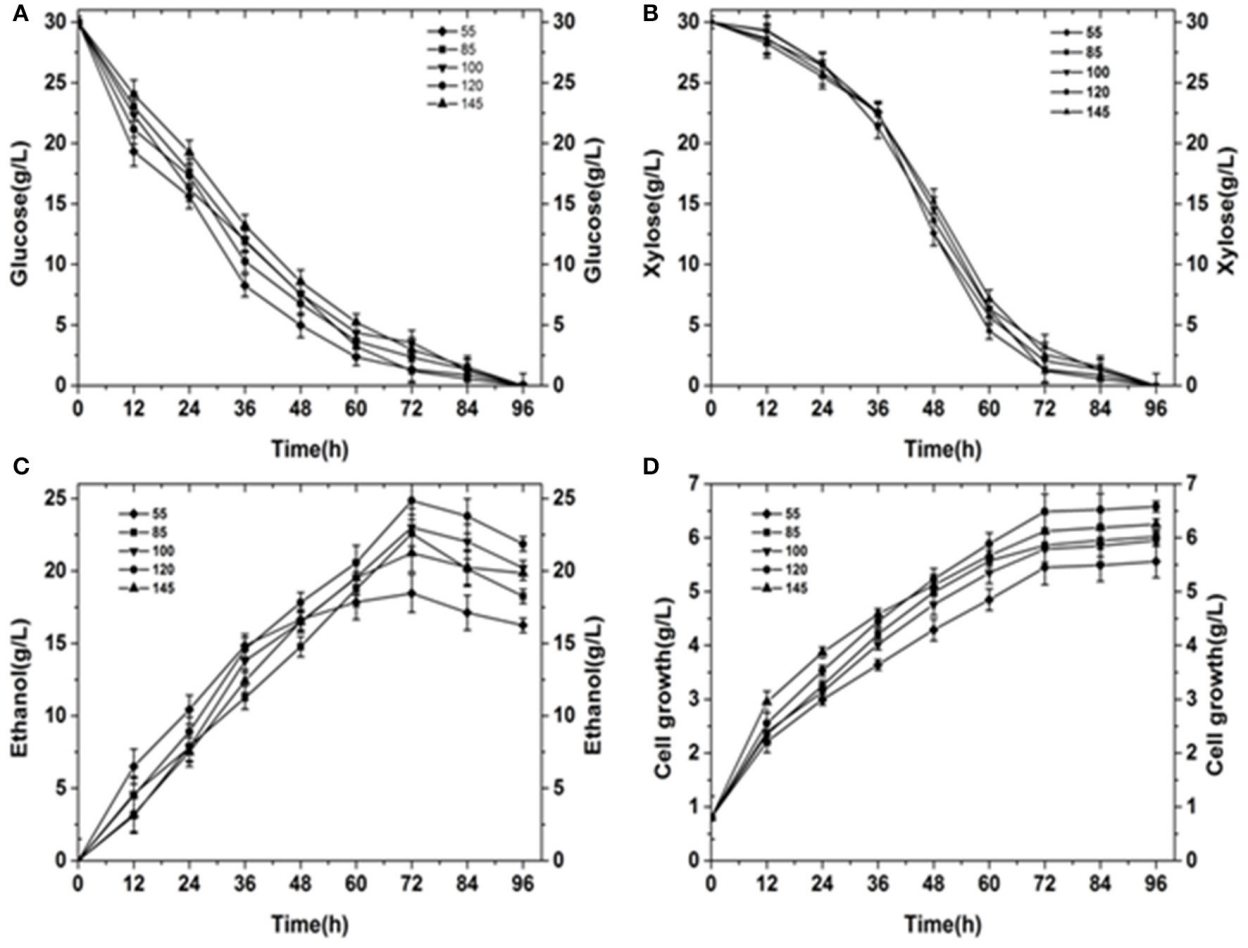
Summary of results obtained under different initial *k*_L_α conditions. **(A)** Glucose consumption under different initial *k*_L_α conditions. **(B)** Xylose consumption under different initial *k*_L_α conditions. **(C)** Ethanol production under different initial *k*_L_α conditions. **(D)** Cell growth under different initial *k*_L_α conditions.

The glucose consumption was showed in Figure 4A. Glucose is the most important carbon source for yeast. Glucose use was most efficient when *k*_L_α was 55. For low *k*_L_α values, yeast cannot carry out normal aerobic metabolism, and anaerobic respiration is necessary to maintain life activity. However, the anaerobic respiration capacity is small and accordingly, large amounts of glucose must be consumed. When *k*_L_α is 145, the sugar utilization efficiency is at its slowest. Overall, before 60 h, glucose consumption was fast. At 60 h, the glucose content was only 12.5–25% of the initial sugar content. At 72 h, fermentation was close to the endpoint for *k*_L_α values of 55 and 120. The amount of residual sugar was almost zero. However, the residual sugar content for the remaining *k*_L_α conditions was close to 10% near the end of the fermentation period at 84 h.

The change of xylose content was showed in Figure 4B during the fermentation process. The use of xylose is a major problem in ethanol fermentation. Wild strains utilizing xylose for ethanol fermentation are rare. Additionally, the efficiency of xylose alcohol production is typically very low. In our analysis, the rate of xylose use in the first 36 h was very slow. In the presence of glucose, yeast first uses glucose, before using xylose. Between 36 and 60 h, the consumption of xylose was very fast. At this point, xylose became the main source for yeast fermentation. The rate of xylose utilization was similar for different *k*_L_α conditions. At 72 h, near the end of the fermentation period, the amount of residual xylose sugar was less than 8% (Fernández-Sandoval et al., 2017).

Ethanol titer during the fermentation process was showed in Figure 4C. Ethanol was the target product. The maximum ethanol titer was obtained when *k*_L_α was 120. The maximum ethanol titer achieved was 24.85 g·L^−1^, which was obtained at 72 h. The conversion rate of sugar to alcohol was 0.447 g·g^−1^ (the theoretical titer of yeast-metabolized xylose was 0.46 g ethanol/g xylose while the ethanol titer for glucose fermentation was 0.51 g ethanol/g glucose; (Ranganathan et al., 2017). The lowest ethanol titer was 18.45 g·L^−1^ at a *k*_L_α of 55 (72 h). For a lower agitation speed and aeration rate, yeast growth was poor and alcohol production was low. However, irrespective of the initial *k*_L_α conditions, final fermentation ended near 72 h. After 72 h, the ethanol content decreased because the yeast required energy to maintain growth. The amount of residual sugar was almost zero, so all of the yeast consumed some ethanol to obtain energy.

Cell weight curve during the process of fermentation was showed in Figure 4D. Cell growth was fastest for a *k*_L_α of 120 and slowest for a *k*_L_α of 55. Although the rate of cell growth was different for different *k*_L_α values, the rate of growth in the first 12 h was significantly higher than that after 12 h, because at 12 h the yeast was in the logarithmic growth phase. The maximum cell weight was 6.49 g·L^−1^ for an initial *k*_L_α of 120, and this was significantly higher than the maximum cell weight under other *k*_L_α conditions.

### Dissolved oxygen analysis

In the case of different initial *k*_L_α values, the initial dissolved oxygen amount was set to 100%. We then followed the change of dissolved oxygen over time for different values of *k*_L_α.

**Figure 5:**
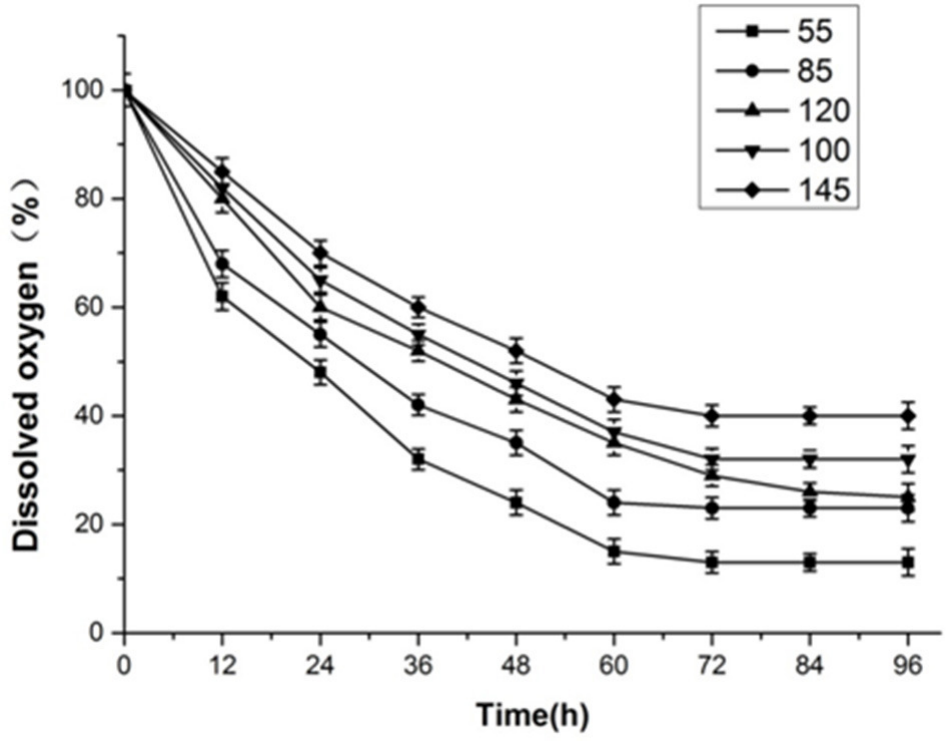
Dissolved oxygen curve.

It can be seen from Figure 5 that the amount of dissolved oxygen clearly declined in the first 60 h, and later stabilized. The amount of dissolved oxygen is important for the fermentation of yeast, directly affecting the fermentation stability, and is an important parameter of fermentation control. Aerobic respiration occurs with appropriate amounts of dissolved oxygen, allowing the yeast to produce more energy that accelerates growth and metabolism. Below the critical amount of dissolved oxygen, anaerobic respiration (fermentation) occurs, resulting in ethanol and other metabolites. With a large number of yeast breeding, oxygen demand grows and the dissolved oxygen concentration in the tank decreases. Since the fermentation condition is best when *k*_L_α is 120, a set of experimental results was obtained by simulating the change in dissolved oxygen by controlling the agitation and aeration coupling.

**Figure 6:**
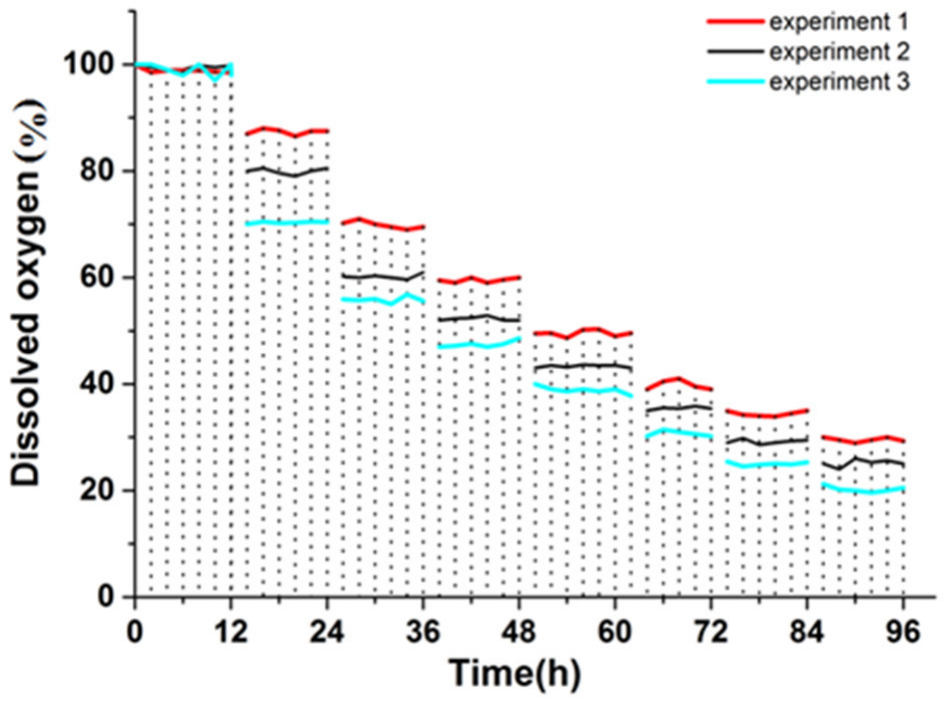
Dissolved oxygen curve.

Figure 6 showed that three sets of experimental values were obtained by three sets of simulation experiments. The first group of experimental ethanol production was 22.56 g·L^−1^ (72 h), the second group was 26.56 g·L^−1^ (72 h), and the third group was 23.85 g·L^−1^ (72 h).

**Figure 7:**
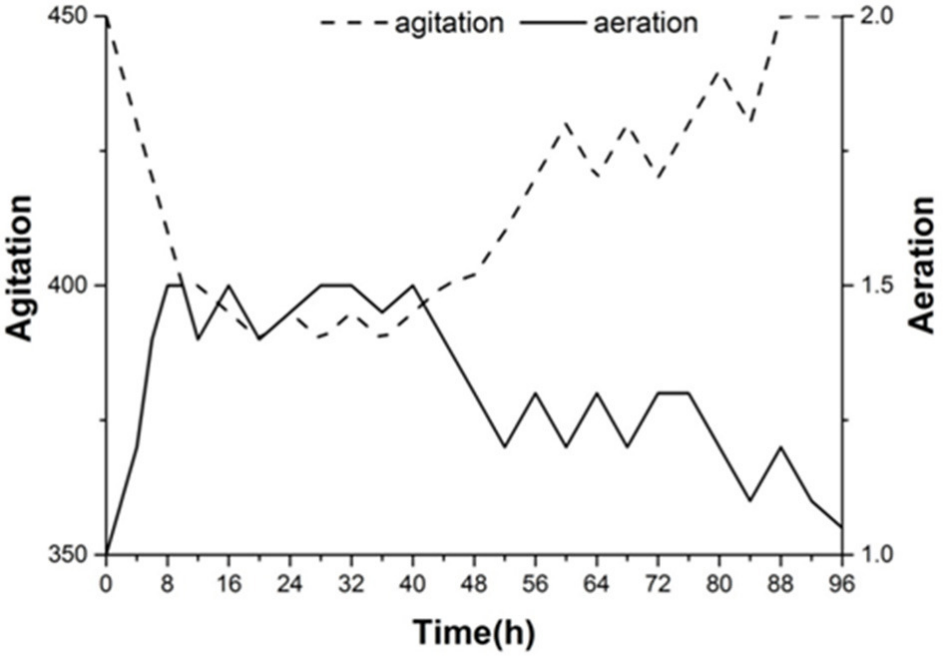
Dissolved oxygen curve for agitation and aeration coupling conditions of the second experimental group.

Figure 7 shows the agitation and aeration coupling curve for the second group of experiments. To control the amount of dissolved oxygen at different stages of the fermentation process, we used the coupling of agitation and aeration to adjust the oxygen content. We can see that increased aeration and decreased agitation helps increase the dissolved oxygen in the early stage, which was beneficial for yeast growth. With the decline in dissolved oxygen in the medium, aeration was gradually decreased to 1.5 L·min^−1^, and the agitation rate was increased slowly. The agitation rate increased by nearly 450 r·min^−1^ and aeration was reduced to 1.0 L·min^−1^ in the later stage of fermentation. During this period, it was beneficial to decrease the viscosity of the fermentation broth and increase the fermentation.

### Comparative fermentation analysis of wild type strain and mutant strain (*k*_L_α = 120)

**Figure 8:**
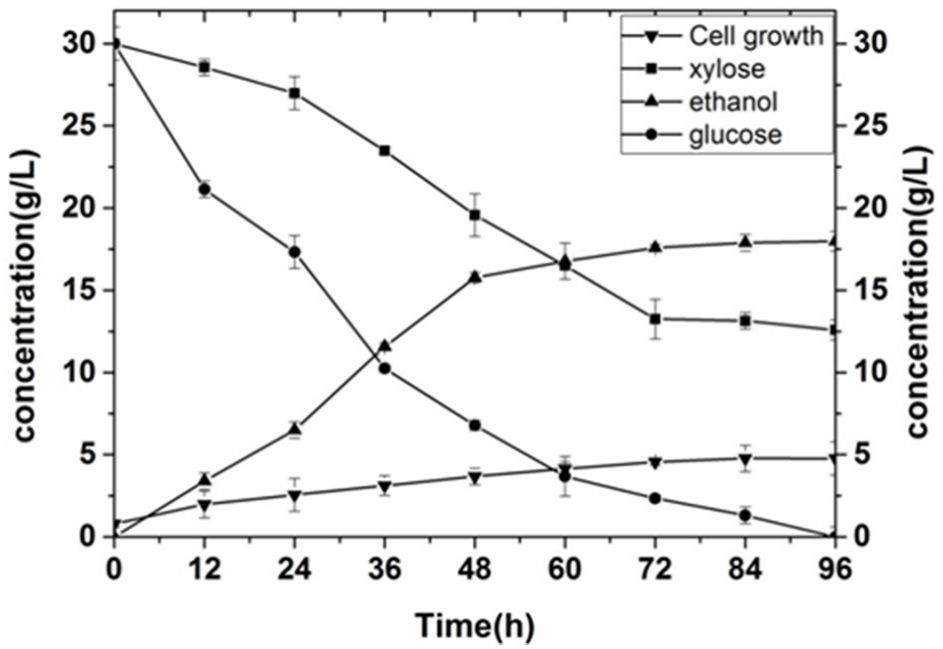
Fermentation of the wild type strain.

In order to compare the productivity of the mutant with that of the wild type strain, we analyzed the main parameters, and Figure 8 showed the fermentation of the original yeast strain at a *k*_L_α of 120. For the wild type stain, glucose utilization was better with a utilization rate of nearly 95%, but the use of xylose was poor. The residual sugar content of xylose was close to 13.25 g·L^−1^ after 72 h of fermentation, and the utilization rate of xylose only reached 55.8%. This resulted in an ethanol production of only 17.58 g·L^−1^. However, the ethanol production of mutagenic screened yeast at a *k*_L_α of 120 was 24.85 g·L^−1^, with a utilization rate of glucose of 92%, and a utilization rate of xylose of 91%. These results indicate that the mutant yeast strain exhibits greatly improved xylose consumption compared to the wild type stain.

### Metabolic flux analysis

After studying the effects of different fermentation processes on ethanol production, metabolic reaction and related enzymes were analyzed in Table 4, the metabolic flow chart was constructed and the metabolic equations were listed. The flow rate of each metabolic point was calculated using the matrix and subsequently analyzed. In order to clarify metabolism of the strains, we analyzed the metabolic pathway and its flux.

**Table 4:** Metabolic reaction and related enzymes.

**No**.	**Enzyme**	**Metabolic reactions**
1	Glucokinase	ATP+Glucose = ADP+Glucose-6P
2	Glucose-phosphate-isomerase	Glucose-6P = Fructose-6P
3	Phosphofructokinase	ATP+Fructose-6P = ADP+Fructose-1,6-2P
4	Fructose-bisphosphate-aldolase	Fructose-1,6-2P = 2 × G3P
5	Phosphoglycerate-k = kinase	G3P+NAD^+^+Pi+ADP = PEP+NADH+ATP+H_2_O+H^+^
6	Xylose reductase	NADPH+Xylose = Xylitol+NADP^+^
7	Xylose dehydrogenase	Xylitol+NAD^+^ = Xylulose+NADH
8	Xanthine kinase	ATP+Xylulose = ADP+5-phosphate xylulose
9	Glucose-6-phosphatedehydro genase	Glucose-6P+2NADP^+^ = 2NADPH+Ribulose-5P+CO_2_
10	Ribose 5-phosphate isomerate	Ribulose-5P = Ribose-5P
11	Transketolase	Ribose-5P+ ylulose-5P = Sep-7P+G3P
12	Transaldolase	Sep-7P+G3P = Eyr-4P+Fructose-6P
13	Transketolase	Eyr-4P+Xylulose-5P = G3P+Fructose-6P
14	Ribulose-phosphate-3-epimerate	Xylulose-5P = Ribulose-5P
15	Pyruvate-kinase	ADP+PEP = ATP+Pyruvate
16	Pyruvate carboxylase	Pyruvate+CoA+NAD^+^ = Acetyl-CoA+CO_2_ +NADH
17	Pyruvate dehydrogenase	ATP+Pyruvate+${\text{HCO}}_{3}^{-}=$ADP+Pi+OAA
18	Citrate synthase	Acetyl-CoA+H_2_O+OAA = Citrate+CoA
19	Aconitine synthase	Citrate = Isocitrate
20	Isocitrate dehydrogenase	Isocitrate+NAD^+^ = α-K+CO_2_+NADH
21	Succinate dehydrogenase	FAD^+^+Fumarate = Succinate+FADH
22	Fumarase	Fumarate+H_2_O = L-malic acid
23	Malate dehydrogenase	Malate+NAD^+^ = OAA+NADH+H^+^
24	Pyruvate decarboxylase	Pyruvate = Acetaldehyde +CO_2_
25	Alcohol dehydrogenase	Acetaldehyde+NADH = Ethanol+NAD^+^
26	Acetyl coenzyme	Acetaldehyde+NAD^+^ = Acetic acid+NADH
27	Aldehyde dehydrogenase	Acetic acid+CoA+2ATP = Acetyl-CoA+2ADP+2Pi

**Figure 9:**
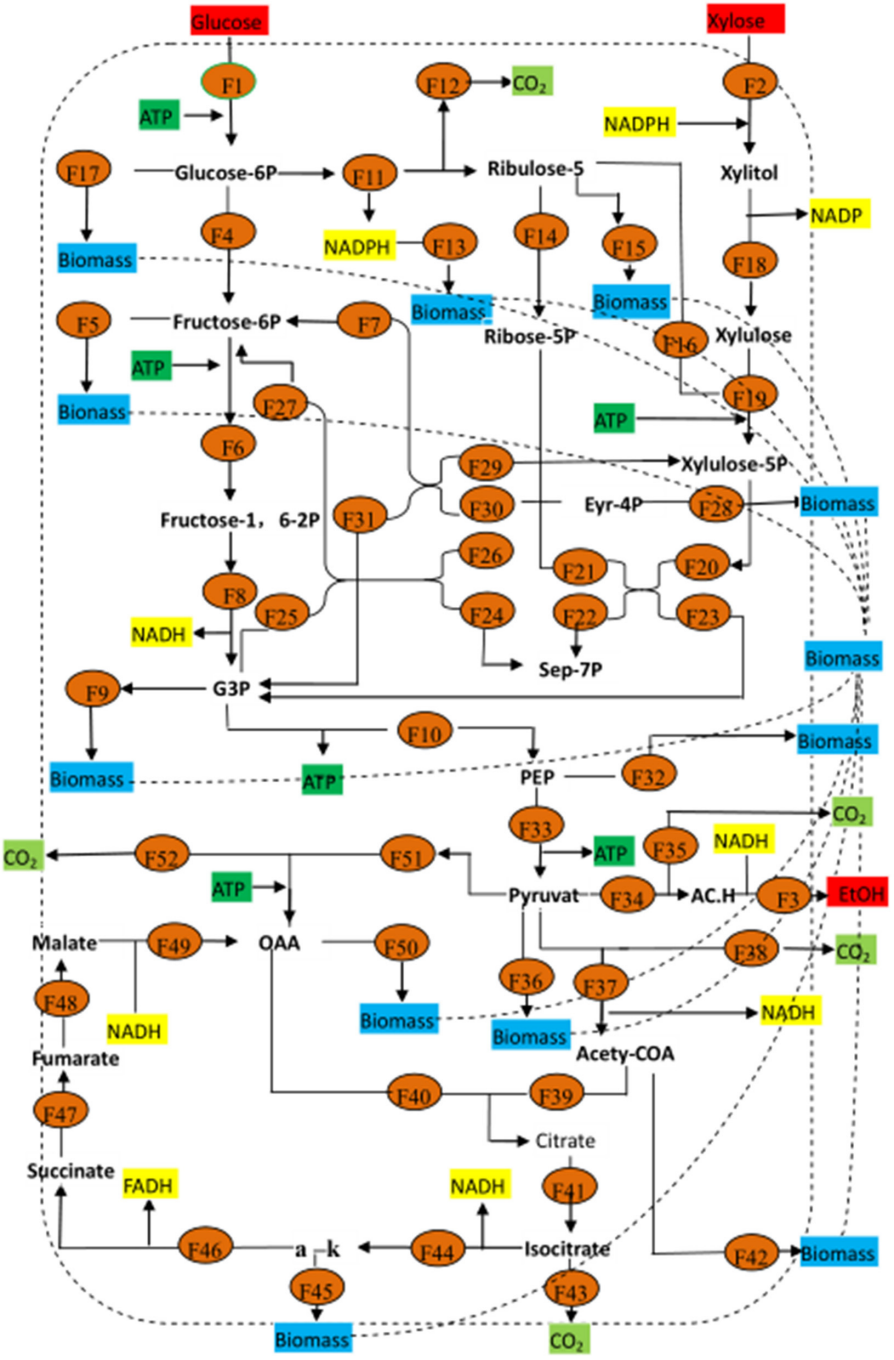
Metabolic flux for *Candida tropicalis CICC1779–Dyd*. All the word Biomasshere refers to the biological composition on the cell. Glucose-6P, 6-phosphate dextrose; Fructose-6P, fructose 6 phosphate; Fructose-1, 6-2P Fructose-1, 6-2 phosphate; Ribulose-5, 5 phosphate ribuolse; Ribulose-5P, 5 phosphate ribose; Eyr-4P, 4 phosphate erythritol; Sep-7P, 7 phosphate Jing Tian heptanose sugar; G3P, glyceraldehyde 3-phosphate; PEP, phosphoenolpyruvate; a-k, α-keto-glutaric acid; AC.H, Acetaldehyde; Etoh, ethanol.

Figure 9 shows the metabolic flow chart of yeast, and Tables 5, 6 list 53 flow equations and their flow values. While equations 1–39 are based on the bioreactivity of the mass balance of the base material, the coefficient constants of equations 39–50 are based on past studies (Longacre et al., 1997; McKinlay et al., 2007). Substrate input, product output, and cell production were directly detected. The cell biomass was nearly fixed in the range of 60–72 h, and consequently we chose this period for our flux analysis. The total throughput of the system had 53 metabolites corresponding to 53 responses, which were calculated by the matrix of these fluxes, the results of which are shown Table 6.

**Table 5:** Flux equations for the *Candida tropicalis* DYD-007.

**No**.	**Metabolic**	**Flux equations**
1	Glucose-6P	1 F_1_−F_4_−F_17_−F_11_ = 0
		2 F_11_−F_12_ = 0
2	Fructose	3 F_4_+F_7_+F_27_−F_5_−F_6_ = 0
3	Fructose-1,6−2P	4 F_6_−0.5 × F_8_ = 0
4	G3P	5 F_8_+F_23_+F_31_−F_9_−F_10_−F_25_ = 0
5	Xylitol	6 F_2_−F_18_ = 0
6	Xylulose	7 F_18_−F_19_ = 0
7	Xylulose-5P	8 F_16_−F_19_+F_20_+F_29_ = 0
8	Ribose-5P	9 F_14_−F_21_ = 0
		10 F_22_−F_24_ = 0
9	Sep−7P and Eyr−4P	11 F_26_−F_28_−F_30_ = 0
		12 F_20_−F_21_ = 0
		13 F_22_−F_23_ = 0
		14 F_20_−F_23_ = 0
		15 F_24_−F_25_ = 0
		16 F_26_−F_27_ = 0
		17 F_24_−F_26_ = 0
		18 F_29_−F_30_ = 0
		19 F_7_−F_31_ = 0
		20 F_30_−F_31_ = 0
10	Ribulose-5P	21 F_11_−F_16_−F_14_−F_15_ = 0
11	PEP	22 F_10_−F_32_−F_33_ = 0
12	Pyruvate	23 F_33_−F_34_−F_36_−F_37_−F_51_ = 0
		24 F_37_−F_38_ = 0
13	OAA	25 F_49_+F_51_−F_50_−F_40_ = 0
		26 F_51_−F_49_ = 0
		27 F_51_−F_52_ = 0
14	Acetyl-CoA	28 F_37_−F_39_−F_42_ = 0
15	Citrate	29 F_39_+F_40_−F_41_ = 0
		30 F_39_−F_40_ = 0
16	Isocitrate	31 F_41_−F_44_ = 0
		32 F_43_−F_44_ = 0
17	α−k	33 F_44_−F_45_−F_46_ = 0
18	Succinate	34 F_46_−F_47_ = 0
19	Fumarate	35 F_47_−F_48_ = 0
20	Malate	36 F_48_−F_49_ = 0
21	NADPH	37 F_2_−2 × F_11 =_0
22	Ethanol	38 F_34_−F_35_−F_3_ = 0
		39 F_35_−F_3_ = 0
25	BM synthesis	40 320.02 × F_5_ = ΔBM
		41 27.45 × F_9_ = ΔBM
		42 3.00 × F_13_ = ΔBM
		43 58.78 × F_15_ = ΔBM
		44 98.35 × F_17_ = ΔBM
		45 165.26 × F_28_ = ΔBM
		46 76.37 × F_32_ = ΔBM
		47 14.59 × F_38_ = ΔBM
		48 26.85 × F_52_ = ΔBM
		49 13.41 × F_41_ = ΔBM
		50 134.41 × F_47_ = ΔBM
26	Substrate uptake	51 F_2_ = ΔXylose
27	Product output	52 F_3_ = ΔEthanol
28	Biomsass	53 F_53_ = ΔBiomsass

**Table 6:** Results of MFA.

**Flux data**	**a**	**b**	**c**	**d**	**e**	**Flux data**	**a**	**b**	**c**	**d**	**e**
F_1_	0.698	1.199	1.299	0.819	1.119	F_28_	0.003	0.004	0.004	0.003	0.004
F2	0.225	0.035	0.021	0.062	0.034	F_29_	0.192	0.131	0.128	0.111	0.124
F_3_	0.973	1.376	1.471	0.972	1.287	F_30_	0.192	0.131	0.128	0.111	0.124
F_4_	0.581	1.174	1.281	0.783	1.096	F_31_	0.192	0.131	0.128	0.111	0.124
F_5_	0.002	0.002	0.002	0.001	0.002	F_32_	0.061	0.087	0.090	0.062	0.082
F_6_	0.967	1.438	1.538	1.007	1.345	F_33_	2.047	2.895	3.089	2.046	2.709
F_7_	0.192	0.131	0.128	0.111	0.124	F_34_	1.947	2.752	2.941	1.945	2.574
F_8_	1.934	2.876	3.0767	2.013	2.690	F_35_	0.973	1.376	1.471	0.972	1.287
F_9_	0.017	0.024	0.025	0.017	0.023	F_36_	0.032	0.046	0.047	0.032	0.043
F_10_	2.109	2.982	3.179	2.107	2.791	F_37_	0.048	0.068	0.070	0.048	0.064
F_11_	0.113	0.017	0.011	0.031	0.017	F_38_	0.048	0.068	0.070	0.048	0.064
F_12_	0.113	0.017	0.011	0.031	0.017	F_39_	0.012	0.017	0.018	0.012	0.016
F_13_	0.156	0.222	0.230	0.157	0.210	F_40_	0.012	0.017	0.018	0.012	0.016
F_14_	0.195	0.135	0.132	0.114	0.128	F_41_	0.024	0.035	0.036	0.024	0.033
F_15_	0.080	0.113	0.117	0.080	0.107	F_42_	0.035	0.050	0.052	0.036	0.048
F_16_	0.162	0.231	0.238	0.163	0.218	F_43_	0.024	0.035	0.036	0.024	0.033
F_17_	0.005	0.007	0.007	0.005	0.006	F_44_	0.024	0.035	0.036	0.024	0.033
F_18_	0.225	0.035	0.021	0.062	0.034	F_45_	0.004	0.005	0.005	0.003	0.005
F_19_	0.225	0.035	0.021	0.062	0.034	F_46_	0.021	0.03	0.031	0.021	0.028
F_20_	0.195	0.135	0.132	0.114	0.128	F_47_	0.021	0.03	0.031	0.021	0.028
F_21_	0.195	0.135	0.132	0.114	0.128	F_48_	0.021	0.03	0.031	0.021	0.028
F_22_	0.195	0.135	0.132	0.114	0.128	F_49_	0.021	0.03	0.031	0.021	0.028
F_23_	0.195	0.135	0.132	0.114	0.128	F_50_	0.017	0.025	0.026	0.018	0.023
F_24_	0.195	0.135	0.132	0.114	0.128	F_51_	0.021	0.03	0.031	0.021	0.028
F_25_	0.195	0.135	0.132	0.114	0.128	F_52_	0.021	0.03	0.031	0.021	0.028
F_26_	0.195	0.135	0.132	0.114	0.128	F_53_	0.469	0.667	0.689	0.471	0.629
F_27_	0.195	0.135	0.132	0.114	0.128						

*“a” represents the metabolic flux of the wild type strain under k_L_α 120; “b” represents the metabolic flux of the mutant strain at k_L_α 120; “c” represents the metabolic flux of the mutant strain coupled with the agitation rate at the agitation rate; “d” represents the metabolic flux from fermentation of the hydrolyzate of the non-detoxified cassava residue; and “e” represents the metabolic flux from the detoxified cassava residue hydrolyzate. Red color/F16 represents a reversible pathway with negative value. And all the metabolic flux units are mmol/(g DWh)*.

Changes in input substrate and product flux were consistent with changes in glucose and xylose consumption as well as ethanol production. Table 6 shows the metabolic flux data at certain condition of the wild type stain and the mutant strain. It is clear that the metabolic flux of the wild type stain is less than in other conditions, especially for the metabolism of xylose. The mutagenic strain's ability to metabolize xylose was significantly enhanced. By analyzing the influence of dissolved oxygen on the metabolic flux, it was found that increased aeration and decreased agitation in the early stage was beneficial for increasing the dissolved oxygen concentration in the tank. The respiratory metabolism of the yeast produced a lot of energy for self-growth, which was greater than the cell oxygen demand. This made conditions suitable for the respiratory metabolism of yeast to produce significant energy for its own breeding. When dissolved oxygen is greater than the cell oxygen demand, yeast undergoes aerobic respiration and metabolic materials cycle more from pyruvate into the tricarboxylic acid cycle to produce a large amount of energy that is used to maintain cell growth. Increasing agitation and decreasing the aeration rate is beneficial in the late stages. Air entering the fermentation broth is dispersed in small bubbles to increase the gas-liquid contact area and strengthen the turbulence of the fermentation broth, reducing the mycelia knot. During this period, the amount of dissolved oxygen is less than the cell oxygen demand, and the yeast undergoes anaerobic respiration, where the material circulates more through pyruvate and acetaldehyde metabolism to yield ethanol.

### Fermentation of hydrolyzed sugar from cassava residue

The highest final yield of ethanol reached was 24.85 g·L^−1^ at 72 h when *k*_L_α was 120. Based on this, we further used cassava residue hydrolyzate for fermentation.

The final concentration of glucose 34.25 g·L^−1^, xylose 25.20 g·L^−1^ were obtained from hydrolyzate from cassava residue. The ventilation and stirring rate were adjusted, so that the initial *k*_L_α was 120. After 72 h of fermentation an ethanol concentration of 23.24 g·L^−1^ was obtained from the detoxified hydrolyzate and its flux was also considerably high, which is 1.287 mmol/(g DWh) in Table 6. For the non-detoxified hydrolyzate, the final ethanol concentration obtained was 17.56 g·L^−1^. Detoxification was necessary as the cassava residue hydrolyzate contains cyanide and acidic substances, which will inhibit the growth and reproduction of yeast, thus affecting the ethanol yield.

## Conclusions

A yeast strain was screened by UV mutagenesis, and fermentation was subsequently carried out under different initial liquid phase oxygen transfer coefficients (*k*_L_α). Compared to the wild type stain, the mutant yeast exhibited improved xylose metabolism. The experimental results show that the fermentation efficiency is highest when *k*_L_α is 120, leading to an ethanol yield of 24.85 g·L^−1^ after 72 h, which is higher than the original yeast strain, reflecting the efficient use of xylose. After optimizing the conditions of the coupled control of aeration rate and agitation speed to obtain the desired change in dissolved oxygen, ethanol production reached 26.56 g·L^−1^. Through the detection of enzyme activity, a metabolic flow chart and equations were constructed. The analysis of metabolic flux shows that dissolved oxygen has a great impact on fermentation. Thus, through these studies we have a better understanding of the metabolic mechanism of yeast and fermentation conditions, laying the foundation for the industrialization of large quantities of ethanol production.

## Author contributions

XL designed the study and wrote the protocol. YD conducted all experiments. YY, ZW, and JC took part in materials preparation. LC, DM, and SL took part in experiments. ZZ and SJ analyzed data. XW wrote and revised the manuscript.

### Conflict of interest statement

The authors declare that the research was conducted in the absence of any commercial or financial relationships that could be construed as a potential conflict of interest.
